# From rejection to vasculopathy: a review of non-invasive biomarkers in alloimmune responses in heart transplantation

**DOI:** 10.3389/fmed.2026.1923304

**Published:** 2026-09-23

**Authors:** Paula Majó Ribera, Lucy Bates, William Scott, Andrew Fisher, Oscar Gonzalez-Fernandez

**Affiliations:** 1 Translational and Clinical Research Institute, Newcastle University, Newcastle upon Tyne, United Kingdom; 2 Institute of Transplantation, Newcastle Upon Tyne Hospitals NHS Foundation Trust, Newcastle upon Tyne, United Kingdom

**Keywords:** biomarker, cardiac allograft vasculopathy (CAV), cell free DNA (cfDNA), donor specific antibodies, gene expression profile (GEP), heart transplantation (HTx), microRNA

## Abstract

**Background:**

Heart transplantation is the gold standard treatment for end-stage heart failure. Despite immunosuppression as the cornerstone therapy for post-transplant management, the presence of immunological complications including acute cellular rejection (ACR), antibody-mediated rejection (AMR), and cardiac allograft vasculopathy (CAV)—remain major barriers to long-term graft survival. Graft monitoring largely remains dependent on protocolized endomyocardial biopsy (EMBx), an invasive, risky procedure.

**Scope:**

This narrative review focuses on aggregating the published evidence, summarizing key clinical complications, and presenting the available tools to date. Furthermore, we discuss key limitations which must be addressed to improve patient care, including the need for non-invasive biomarkers in cardiac allograft vasculopathy

**Key findings:**

Significant progress in the development and promising use of non-invasive biomarkers for the detection of graft rejection has emerged over the last decade; particularly for monitoring ACR and AMR. Donor-derived cell-free DNA and gene expression profiling of circulating immune cells have both emerged as promising tools to aide post-transplant management of graft health and reduce EMBx frequency. Soluble protein biomarkers—including hs-cTn, BNP, and NT-proBNP—provide supplementary information on graft stress and injury but lack specificity. In contrast, evidence supporting non-invasive biomarker use in CAV remains sparse.

**Conclusions:**

Non-invasive biomarkers have meaningfully advanced rejection surveillance in heart transplantation and hold promise for reducing procedural burden on patients. However, their role in detecting and monitoring CAV remains underdeveloped, and represents the most critical unmet need in the field.

## 1 Introduction

Heart transplantation is the most effective treatment for eligible patients with end-stage heart failure. A population with a greater burden of comorbidities has led to a steady rise in demand for transplantation; with more than 5,800 procedures performed globally each year. Steady progress has led to short-term success for this intervention, with one-year post-transplant survival rates reaching 92.1% between 2018 and 2023 and improvements to quality of life (1). To further improve patient mortality outcomes, there needs to be a better understanding of the mechanistic drivers that modulate the different types of cardiac allograft loss and patient death, despite a 12.5-year increase in median survival following HTx (2).

Immediately after a heart transplant (HTx) primary clinical focus is patient survival, with the leading cause of 30-day mortality attributed to primary graft dysfunction (PGD). Other post-HTx complications consist of infections, including deep surgical wound infections, rejection, renal failure, arrhythmias, and pericardial effusions (3–5) (Figure 1). Progress has been made to mitigate these early risks through the improvement of donor-recipient matching, improved organ preservation techniques, and pre-transplant medication therapy (2, 6). Following the perioperative period, clinical care shifts focus to long-term management; particularly, rejection and the many complications associated with immunosuppression.

**Figure 1:**
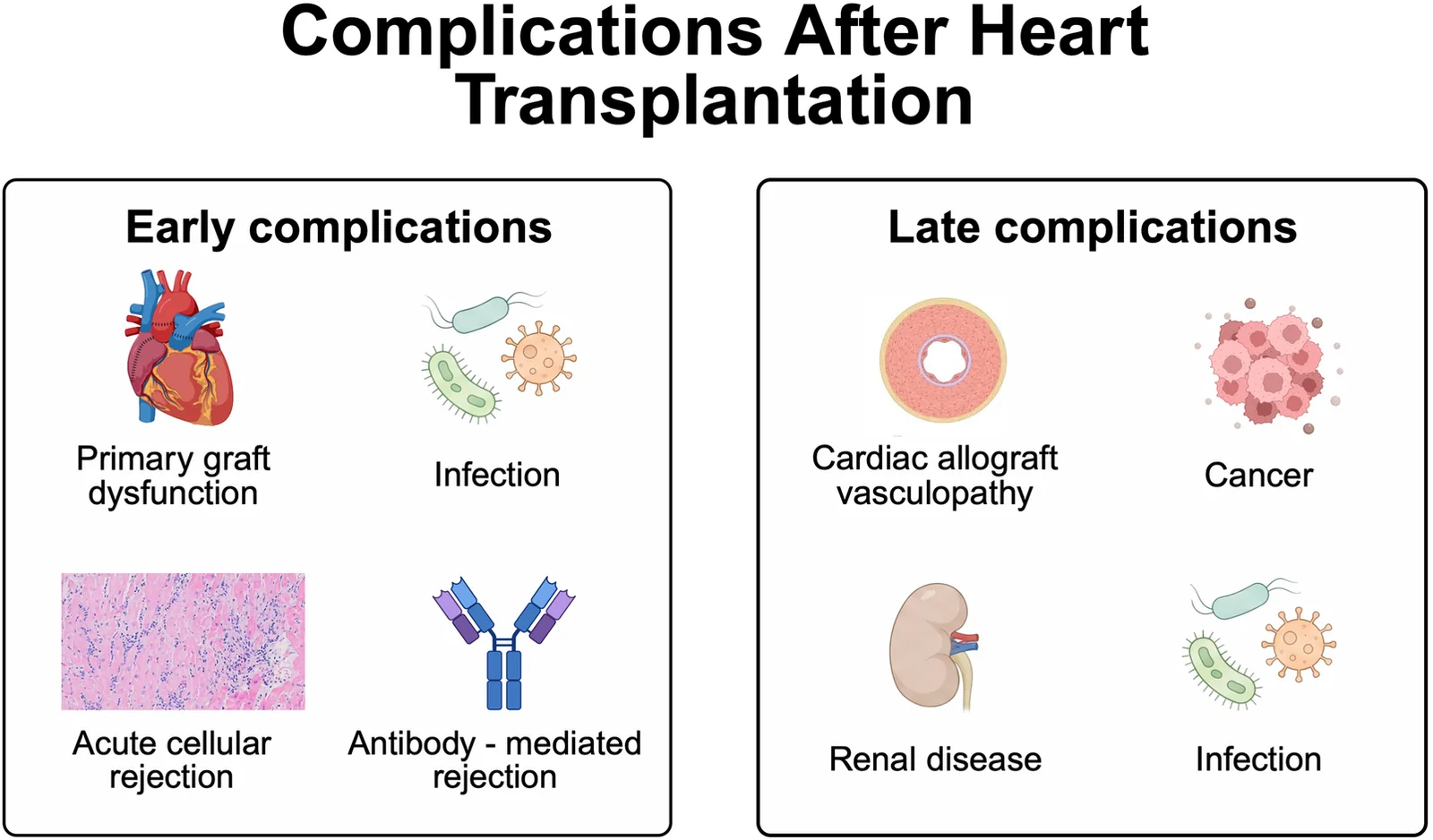
Complications after heart transplantation. Complications after heart transplantation, separated by time of onset. Early complications (left), primary graft dysfunction, infection, acute cellular rejection, and antibody-mediated rejection, prevalent in the first few month's post-transplant, when patient survival is the primary clinical focus. Late complications (right), cardiac allograft vasculopathy, malignancy, renal disease, and infection, emerge over the longer term, driven by factors including lifelong immunosuppression and ongoing alloimmune injury. Figure created in BioRender.

HTx recipients are constrained to lifelong medications. This includes immunosuppression to prevent rejection and long-term treatments, such as acyclovir and co-trimoxazole, to reduce the risk and treat infections (7). As well as routine lipid modifying agents that aid in delaying cardiac allograft vasculopathy (CAV).

Medication non-adherence is one of the key barriers that negatively impacts longer-term survival outcomes. The BRIGHT study, the largest international data set for medication non-adherence in HTx recipients, demonstrated that 37.4% of patients showed non-adherence to their immunosuppression medications over a four-week period (8). By optimizing this modifiable risk factor, improvements in graft longevity could be achieved as it would lead to the reduction of acute rejection (AR) episodes and therefore graft loss and mortality in HTx recipients (9).

After transplant, a variety of pathological mechanisms impact graft health. If left untreated it can result in loss of the graft and subsequent death of the patient; and so, requires lifelong surveillance. Here we present an overview of the main drivers behind this: acute cellular rejection (ACR), antibody-mediated rejection (AMR), and CAV.

There are new and old markers for non-invasive cardiac surveillance. Markers that are established and in use include; brain natriuretic peptide (BNP), high-sensitivity cardiac troponin (hs-cTn), and human leukocyte antigen (HLA) donor-specific antibodies (DSA) (Table 1, Figure 2). hs-cTn provides indirect information on myocardial stress and injury. Whereas DSA signal immune activity and are valuable in the overall diagnosis of AMR.

**Table 1:** Summary table of non-invasive biomarkers used in cardiac allograft surveillance and detection.

Biomarker	Biological rationale	Advantages	Limitations	Guideline recommendation
dd-cfDNA (7, 10)	Donor genome fragments released into plasma by allograft cell injury	ACR and AMR detection	Low PPV Fraction affected by changes in total cfDNA	Recommended by ISHLT guidelines in pandemic settings.
GEP (11)	PBMCs transcriptional signature of alloimmune activation	High NPV ACR	Not validated for AMR	ISHLT guidelines class IIa
DSA (7)	Humoral alloimmunity	Identifies patient at risk for AMR and CAV	Poor sensitivity for AMR No consensus on treating DSAs	ISHLT guidelines recommend routine DSA monitoring
microRNA (12, 13)	Rejection-associated myocardial miRNAs released into plasma	Could distinguish ACR from AMR phenotype	Lack of assay standardization	Investigational only
NT-proBNP (7)	Myocardial wall stretch	Universally available	Non-specific	Adjunctive only
hs-cTn (7, 14)	Cardiomyocyte injury	Ubiquitous, inexpensive	Low specificity	Not recommended for rejection or CAV
sST2 (15, 16)	Myocardial stress	Graded relationship with rejection severity	Marked elevation after bypass, sepsis, and any systemic inflammation	Investigational only
EVs (17, 18)	EVs carry protein/RNA mirroring intra-graft immunopathology	Tissue and cell-type-specific signal	Very small pilot cohort	Investigational only
Cytokines (19)	Inflammatory cytokines released during alloimmune activation	Mechanistically upstream of tissue injury with potential for early detection	Highly non-specific	Investigational only

ACR, acute cellular rejection; AMR, antibody-mediated rejection; CAV, cardiac allograft vasculopathy; dd-cfDNA, donor-derived cell-free DNA; DSA, donor-specific antibody; EVs, extracellular vesicles; GEP, gene expression profiling; hs-cTn, high-sensitivity cardiac troponin; ISHLT, International Society for Heart and Lung Transplantation; miRNA, microRNA; NPV, negative predictive value; NT-proBNP, N-terminal pro-B-type natriuretic peptide; PBMC, peripheral blood mononuclear cell; PPV, positive predictive value; sST2, soluble suppression of tumorigenicity 2.

**Figure 2:**
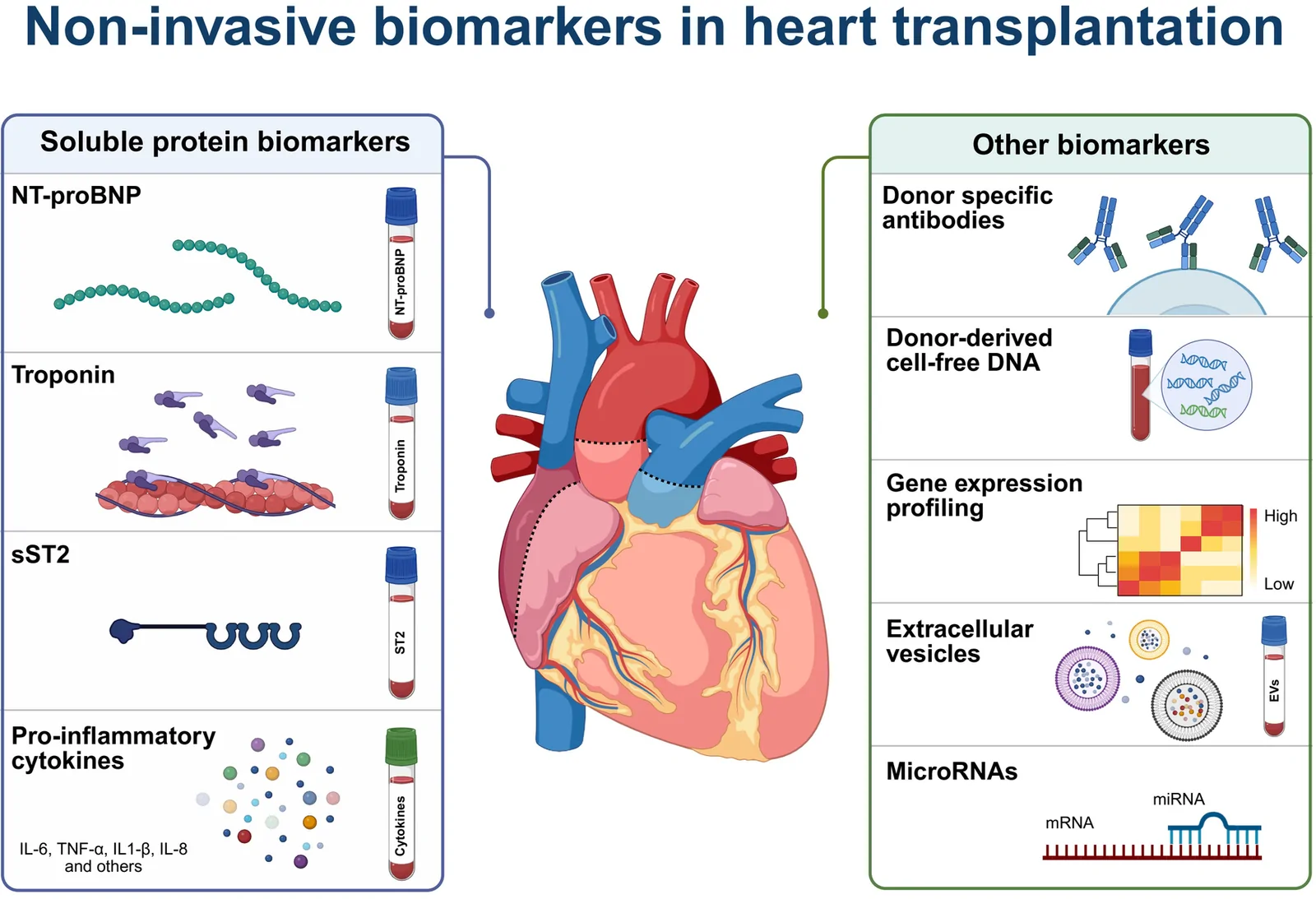
Non-invasive Biomarkers in Heart Transplantation. Biomarkers categorized by class. Soluble protein biomarkers (left), NT-proBNP, troponin, ST2 and pro-inflammatory cytokines (including IL-6, TNF-α, IL-1β, and IL-8), reflect myocardial stress, injury, and systemic inflammation. Other biomarkers (right), donor-specific antibodies, donor-derived cell-free DNA, gene expression profiling, extracellular vesicles (EVs), and microRNAs, reflect the underlying alloimmune response and graft injury directly. Figure Created in BioRender.

However, due to lack of specificity, these markers are not recommended to be used solely in isolation. hs-cTn, has been shown to have high sensitivity and a high negative predictive value (NPV) in the setting of ACR (20). Although there is conflicting evidence with recent studies stating hs-cTn is not suitable for the surveillance or detection of ACR in HTx patients (14). BNP levels can rise due to renal dysfunction, infection, ventricular wall stress, and pressure overload from various pathologies. Therefore, it is not recommended as a standalone marker. DSAs are established in the surveillance and diagnosis of AMR. They also aid in risk stratification of patients who present with *de novo* DSAs, which are associated with adverse outcomes post-HTx. They additionally support the histological diagnosis of AMR and are used to guide immunosuppressive therapy. Overall, these biomarkers would benefit from additional tools such as donor-derived cell-free DNA (dd-cfDNA), gene expression profiling (GEP), and microRNA (miRNA) to enable better graft health monitoring (7).

Endomyocardial biopsy (EMBx) is the current gold standard for the detection and surveillance of AR. Newer non-invasive biomarkers such as dd-cfDNA, GEP, and miRNA are validated against it. However, EMBx is an imperfect reference standard, constrained by sampling error, limited tissue sampling and interobserver variability and, although low occurrence, high-risk complications such as cardiac perforation, severe bleeding, and pneumothorax (21–23). It has been reported that interobserver variability is increased for AMR compared to ACR. Furthermore, patients with elevated dd-cfDNA but a negative biopsy have been shown to go on to develop graft dysfunction, rejection or death in 59% of cases highlighting how EMBx can miss allograft injury and underscoring the need for better tool (24). Therefore, any study design that is compared to an imperfect reference standard cannot achieve 100% sensitivity and specificity.

In addition to the limitations of EMBx as a reference standard, it should also be highlighted that the biomarker assays can be confounded by various clinical factors. These include infection, bleeding, exercise, and hemodynamic compromise (25–27). It is for these reasons that the diagnostic performance reported for the newer non-invasive biomarkers throughout this review should be interpreted with this limitation in mind.

Furthermore, EMBx also poses a mental and fiscal burden on HTx patients. Patients report anxiety due to biopsies; preferring non-invasive procedures (28, 29) Travel also carries a burden, as patients may have to travel far for appointments, possibly creating both financial strain and accessibility barriers (30). This is further highlighted by the fact patients are unable to drive for the first six to eight weeks post-transplant. Limited availability of hospital-patient transport, constraints around public transport timing and accessibility further add to the problem (31). A potential biomarker that could be tested at home may relieve these barriers, allowing for more accessible care.

To truly understand biomarkers for rejection, we must first understand the primary mechanistic drivers that lead to graft loss and how they are treated:

### 1.1 Acute cellular rejection (ACR)

ACR is a T-lymphocyte-mediated immune response that is activated due to the recognition of foreign donor antigens. T-cell activation via direct and indirect pathways leads to the infiltration of cytotoxic T-cells and macrophages into the myocardium, causing irreversible myocyte injury, resulting in disruption of the myocardial architecture eventually leading to allograft dysfunction. This form of rejection is typically diagnosed by means of EMBx, and classified according to the ISHLT classification into four grades 0R, 1R, 2R, and 3R, depending on histopathological findings (Figure 3) (32). It is important to detect ACR promptly as mild to moderate rejection can progress to severe rejection causing damage to the allograft and may lead to an overall reduced chance of survival (33, 34). With ACR affecting over 20% of the HTx population, transplant center protocols typically involve weekly routine biopsies in the first month with the frequency reducing as time after transplant increases (35). Frequent testing is vital as this can allow detection of rejection especially in the first few month's post-transplant where ACR is most prevalent, and adequate immunosuppression can be tailored to aid in the preservation of graft function (7).

**Figure 3:**
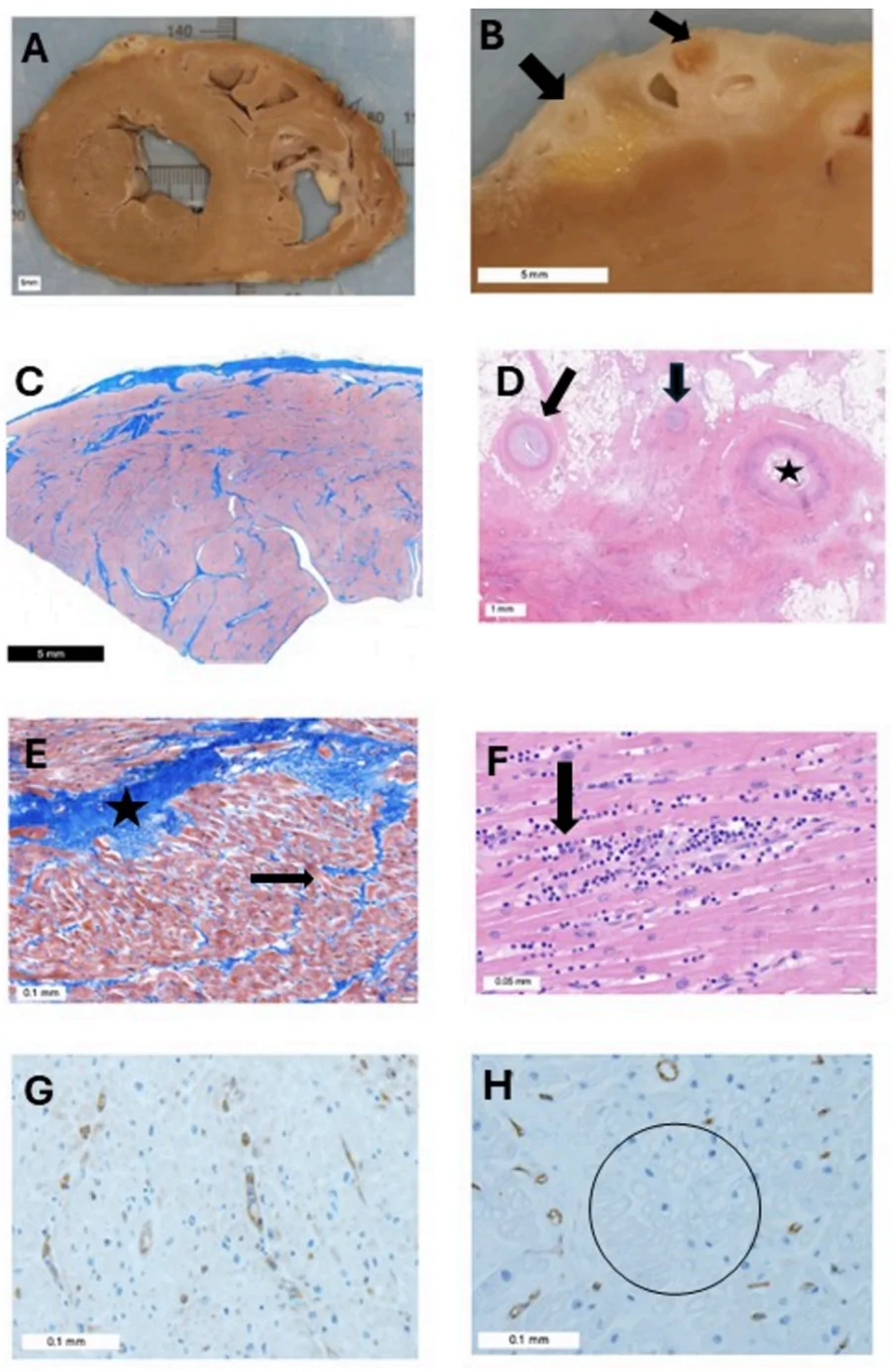
Pathological findings in an explanted heart. **(A)** Short-axis transverse section of the myocardium demonstrating diffuse pallor without overt fibrosis, moderate right ventricular cavity dilatation, and hypertrophy of the left ventricular papillary muscles. **(B)** Macroscopic view at higher magnification of the epicardial coronary arteries (arrows) showing marked luminal narrowing and focal obliteration. **(C)** Full cross-section of the left ventricular wall (MSB stain) revealing mild to focally moderate interstitial fibrosis. **(D)** Histological section (Haematoxylin-Eosin) of epicardial coronary arteries illustrating myointimal proliferation (arrows) with superimposed fibrinous exudate partially occluding the lumen in one of these (star). **(E)** Higher-magnification view (MSB stain) demonstrating the coexistence of fine interstitial fibrosis (arrow) and focal replacement fibrosis (star). **(F)** Focus of acute cellular rejection with associated myocyte injury (arrow). **(G)** C4d immunostaining highlighting multifocal expression within myocardial capillaries, consistent with antibody-mediated rejection. **(H)** CD31 immunostaining revealing an area (circle) of myocardial capillary rarefaction (loss), a morphological correlate of chronic microvascular injury.

### 1.2 Antibody mediated rejection (AMR)

AMR is another form of AR, driven by DSAs binding to HLAs on the endothelium of the transplanted heart, initiating a complex immunological cascade. A hallmark of this process is activation of the complement pathway, generating the split product C4d, which covalently binds on the allograft endothelium and serves both as a mediator of downstream injury and as a diagnostic marker for AMR. C4d deposition is followed by the production of proinflammatory cytokines and chemokines, promoting the recruitment of macrophages, natural killer cells, and other immune cells. Due to complement-dependent and complement-independent pathways, this sustained inflammatory response results in endothelial injury, increased vascular permeability, microvascular thrombosis, and ultimately allograft dysfunction (36). In line with ISHLT guidelines, histological findings including the presence of C4d, clinical assessment of graft function, and the presence of DSA must be collectively evaluated to classify and stratify the risk of AMR (37). Prevalence of AMR varies widely, due to differences in diagnostic criteria across centers and varying sample time points, with some studies reporting 10%–20% and others 40% frequency, reflecting the uncertainty of AMR incidence (38). Nevertheless, it is important to treat AMR as it can lead to graft dysfunction and is associated with reduced long-term survival as well as accelerated progression of CAV (39).

### 1.3 Cardiac allograft vasculopathy (CAV)

CAV is a poorly understood form of chronic rejection that affects epicardial coronary arteries to myocardial capillaries. The pathological hallmark in epicardial coronary arteries is characterized by arterial luminal narrowing caused by T-lymphocyte mediated intimal inflammation and damage resulting in concentric intimal thickening due to myofibroblastic proliferation. Loss of myocardial capillaries and diffuse myocardial fibrosis are also key pathological features leading to graft ischemia (Figure 3) (40).

When disease is found, it is usually already advanced, and graft function is often impaired. There are limited signs and symptoms in early disease due to denervation of the graft during orthotopic HTx surgery, where there is disruption of the parasympathetic and sympathetic autonomic nervous system to the donor heart (41). Consequently, patients commonly develop symptoms only in the last stages of graft dysfunction, delaying clinical recognition. This poses the need for recognition in the early phases of the disease, and routine surveillance is recommended in post-transplant care. In addition, coronary angiography is restricted to the main epicardial vessels and cannot detect abnormalities within the microvasculature and as a result, it may miss early or diffuse small-vessel injury that could be contributing to both the development and severity of CAV (42). The use of intravascular ultrasound during coronary angiography increases detection rates. However, it requires an invasive approach, is limited to the epicardial vessels and is not broadly available as the standard of care (Figure 4) (43). With CAV affecting up to 50% of patients 10 years post-transplant (2), early detection is critical to allow timely initiation of mammalian target of rapamycin inhibitors (mTORi), which have been shown to reduce incidence and slow the progression of CAV (44, 45).

**Figure 4:**
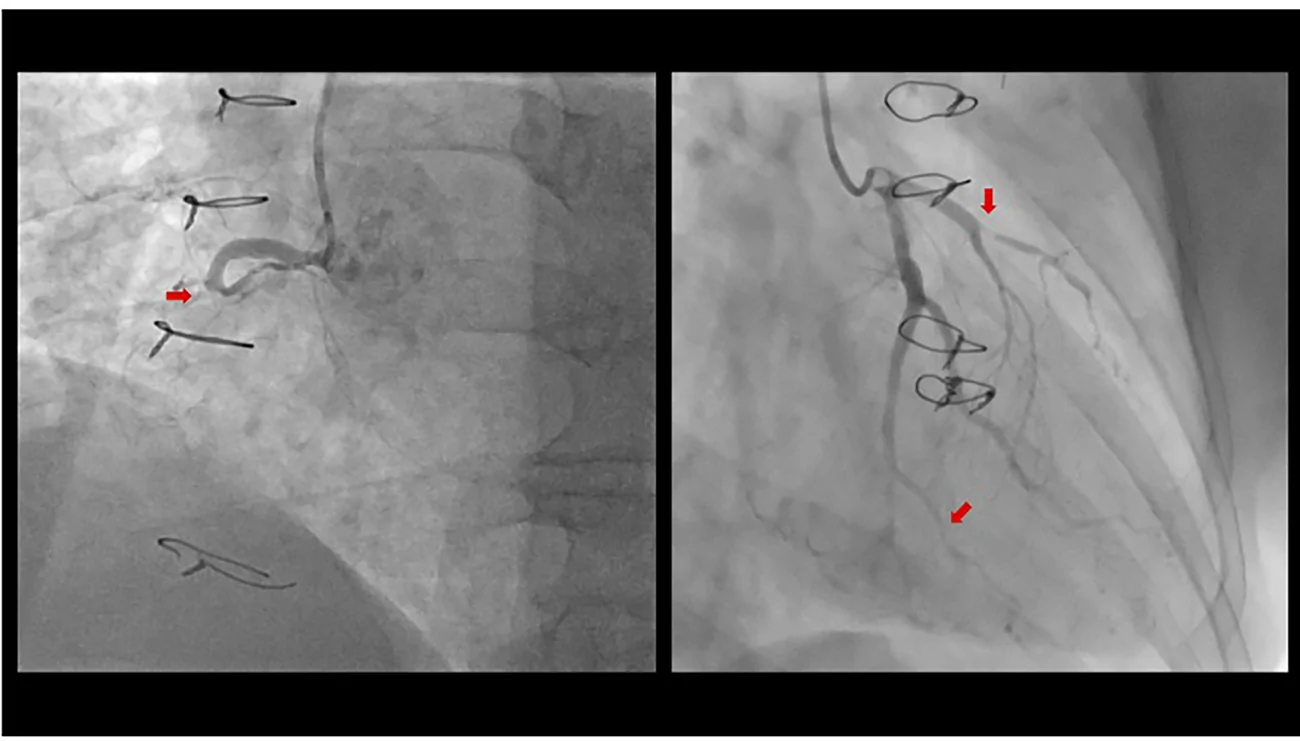
Angiography. Images showing the right coronary artery (left side) and the left system (right side). The right coronary artery has a chronic total occlusion in the mid segment. The left system shows a patent left anterior descending and left circumflex arteries with severe lesions in multiple segments.

The biomarker advances for CAV have lagged acute rejection (AR) research due to the intrinsic mechanisms of both alloimmune responses. CAV is a slowly progressive structural disease not having in most cases an acute event, while rejection is an acute episode with defined histologic criteria for its diagnosis. As a result of these facts, biomarker research in CAV faces significant challenges, and no blood-based biomarkers are currently recommended by the guidelines for CAV surveillance in contrast to AR.

### 1.4 Malignancy, infection and renal complications

Besides the immunological complications in HTx recipients, development of malignancy becomes a significant barrier that patients and clinicians must navigate together. Although immunosuppression therapy remains critical in reducing the risk of rejection, it carries the potential unwanted consequence of over-immunosuppression, which can lead to de-novo cancers due to a diminished immune state (46), as well as an increased risk of infection and sepsis (Figure 5). Clinicians walk a tight rope in calculating immunosuppression treatment. There is a clear need for tools that aid in achieving this balance for improved long-term patient outcomes. Patients are often regularly screened for signs of malignancy, increasing both mental burden for patients and fiscal burden on healthcare systems. Increased risk of infection is also carefully managed and may impact patient socialization as well.

**Figure 5:**
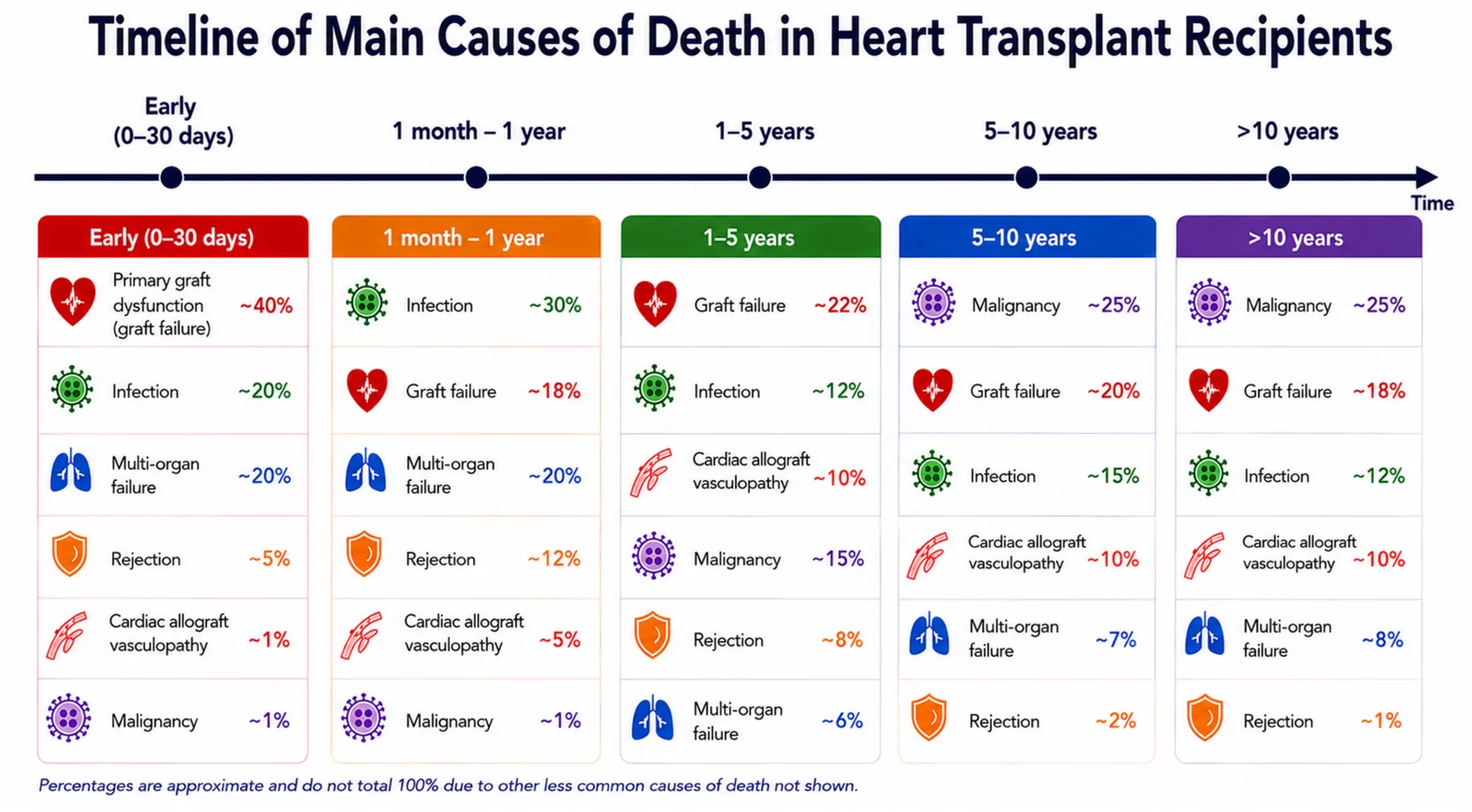
Timeline of main causes of death in heart transplant recipients. Representation of the frequency of causes of death in each period after transplant (0–30 days, 1 month-1 year, 1–5 years, 5–10 years, >10 years) (47–49).

Furthermore, the toxicity profile of long-term immunosuppression treatment also increases the risk of developing chronic kidney disease (50). This underscores the need for novel biomarkers to detect complications earlier and allow for personalized and earlier interventions (47–49).

All biomarkers discussed share a common goal: to identify complications at earlier stages so that management can shift from attempting to rescue a malfunctioning graft to implementing preventative and/or personalized therapy. Despite aiding in earlier detection of disease, it is important to acknowledge their current limitations. Thus far, biomarkers including dd-cfDNA, GEP, and the soluble protein biomarkers are reflective of ongoing graft damage and not indicative of future events or predicting long-term outcomes of allograft health. Nevertheless, they are useful in serving as a timely, non-invasive marker to detect early allograft injury prior to hemodynamic compromise and rejection one month before biopsy-proven AR (51). This may offer clinicians an earlier opportunity to halt disease and allow preservation of long-term graft function and therefore improve patients' mortality and morbidity.

Here, we next focus on non-invasive biomarkers for cardiac allograft surveillance with particular emphasis on their emerging role in the early detection and monitoring of ACR, AMR, and CAV.

## 2 Non-invasive biomarkers

### 2.1 Soluble protein biomarkers

#### 2.1.1 BNP and NT-proBNP

BNP and its inactive cleavage product N-terminal pro-BNP (NT-proBNP), which has a longer half-life, have been investigated as potential biomarkers for allograft surveillance. BNP and NT-proBNP lack specificity to serve as a standalone rejection biomarker after heart transplantation, because it reflects generic hemodynamic and wall-stress stimuli rather than alloimmune-specific injury, and its post-transplant level is confounded by multiple factors. The relationship between ACR and the soluble proteins, BNP, and NT-proBNP varies between studies. Clemmensen and colleagues (52) demonstrated high sensitivity of ACR grade 2R compared to patients without 2R rejection. In studies looking at grade 3R, as the endorsed definition of ACR by ISHLT 2004 guidelines (32), there is low evidence to suggest that BNP or NT-proBNP are appropriate biomarkers to utilize solely in the surveillance and detection of rejection (53).

In AMR, BNP and NT-proBNP are similarly used as secondary biomarkers to aid in the overall diagnosis, and it is not recommended to be used as a singular marker for monitoring rejection in stable HTx patients (7).

For CAV, circulating levels of these peptides have been shown to rise in parallel with disease severity, serving as significant predictors of prevalence and progression (42, 54, 55). However, BNP and NT-proBNP are not specific to CAV; their elevation reflects ventricular wall stress and pressure overload that may also occur as stated above, AMR and ACR, as well as renal dysfunction, or infection (7). Therefore, these peptides should be interpreted within the broader clinical context rather than as standalone diagnostic markers.

#### 2.1.2 High-sensitivity cardiac troponin (hs-cTn)

hs-cTn, levels rise in response to myocardial cellular injury. The hs-cTn assays measure specific isoforms of the troponin complex (T and I) in the blood. Cardiac troponins do not offer diagnostic accuracy as a rejection or vasculopathy test following heart transplantation as they are markers of cardiomyocyte injury from any cause (14).

Regarding ACR, studies show high-sensitivity Troponin (hs-cTnI) to have a sensitivity of 88% and a negative predictive value (NPV) of 92% when compared against biopsy-proven ACR (20). The ISHLT has endorsed the use of hs-cTnI as a useful tool to monitor rejection in high-risk patients, however, it is important to note its limitations of false negative results and low specificity which hinders its ability to be used as a standalone diagnostic and surveillance biomarker for ACR (7).

Patel et al. (56), demonstrated an association between hs-cTnI concentrations and the presence and severity of CAV. Levels of hs-cTnI were approximately four times higher than those without disease, a threshold of 1.5 pg/mL achieved 100% sensitivity and an NPV of 100%, but overall specificity remained low. Although elevated levels of hs-cTnI in this study correlated with poorer long-term outcomes, it did not reliably predict which HTx patients would subsequently go on to develop CAV. Potentially aiding in the differentiation of rejection etiologies a more recent study identified hs-cTnI, hs-cTnT and NT-proBNP to be associated with CAV, when compared to native coronary artery disease where a different set of biomarker profiles were elevated (57).

#### 2.1.3 Novel soluble protein biomarkers

Soluble ST2 (sST2) is the circulating decoy receptor of the IL-33/ST2L axis, released in response to myocardial stretch, fibrosis, and Th2-skewed inflammation, and thus reflects both hemodynamic and immune-mediated graft injury rather than a rejection-specific process. In a cohort of 26 patients, sST2 rose during AR (130 vs. 51 ng-mL; *p* = 0.002) with an AUC of 0.72 and a 68 ng/mL cutoff giving PPV 53% and NPV 83% (OR 4.9, 95% CI 1.7–14.5) (15). Its use is limited as sST2 can increase after cardiopulmonary bypass, and sepsis, making the early post-transplant window uninterpretable (16).

Extracellular vesicles (EV) are membrane-bound nanoparticles carrying cell-of-origin-specific protein and nucleic acid cargo, allowing immunocapture of donor-heart or T-cell-derived vesicles. In 90 patients, EV concentration rose and diameter fell during rejection (*p* < 0.001 for ACR and AMR), and multiplex surface-antigen profiling discriminated phenotypes for ACR (CD3, CD2, ROR1, SSEA-4, HLA-I, CD41b) vs. AMR (HLA-II, CD326, CD19, CD25, CD20) with individual marker AUCs of 0.727 and 0.939 and 86.5% accuracy in external validation (17). Using immunoaffinity-enriched small EVs across 70 time-matched biopsies, donor-heart sEV cTnT protein and mRNA fell while T-cell sEV CD4, CD8, TCR and miRs let-7i, 101b, 21a rose during moderate ACR, and profiles reversed with successful treatment (Spearman 0.85–0.87) (18).

Pro-inflammatory cytokines include circulating TNF-α, IL-1, IL-2, IL-6, and IL-8 are involved in the systemic innate and adaptive immune activation. Infection, CMV reactivation, surgery, and renal dysfunction all elevate these cytokines, and its contemporary use has shifted to combinatorial modeling due to the lack of specificity (19).

### 2.2 Other graft-specific markers

#### 2.2.1 Donor-specific antibodies (DSA)

DSAs are antibodies produced by the recipient's immune system directed against HLA expressed on donor tissue. DSAs mediate graft damage by binding to HLA antigens expressed on the endothelium of the allograft, activating the classical complement cascade. DSAs may also activate and injure endothelial cells via complement-independent pathways (58, 59).

DSAs rarely arise in an immunologically naive host. In many candidates, the humoral alloimmune response is already primed by prior exposure to non-self HLA through pregnancy, transfusions, previous transplantation, homograft material, or mechanical circulatory support, leaving circulating anti-HLA antibodies or memory B-cells capable of a rapid response after re-exposure (24). The calculated panel-reactive antibody (cPRA) estimates the proportion of donor-epitopes against whom a transplant recipient carries HLA antibodies, and sensitizing exposures may increase the cPRA reducing the donor pool (24). In a contemporary single-center cohort of 121 patients, cPRA class I over 50% was significantly associated with AMR, and shorter AMR-free survival (60).

The implantation of the allograft is the triggering event that converts this latent state into clinical disease. At reperfusion, the graft endothelium presents the full donor HLA to a primed recipient, producing immediate antibody binding and complement activation or a memory response within days (58). The presence of preformed, high-titer DSAs may lead to hyperacute rejection within minutes of reperfusion by binding of circulating antibodies to the graft endothelium triggering a complement fixation, and the production of platelet aggregation, thrombotic occlusion, and irreversible necrosis causing graft loss (59).

Due to the etiology of DSAs, they have not proven useful in the surveillance or diagnosis of ACR. DSAs aid in guiding immunosuppression therapy, support the diagnosis of AMR and stratify the risk of patients who present with *de novo* DSA which are associated with adverse outcomes post HTx (7). Clerkin et al. (61) demonstrated the usefulness of DSA at predicting future AMR episodes. Patients who presented with DSAs compared to those with no development of these antibodies were seven times more at risk of having at least one episode of AMR (*p* < 0.0001). The research also identified an increased risk of allograft loss in patients who developed de-novo DSAs (*p* = 0.027). DSAs support the histological AMR diagnosis, and DSA quantification is recommended in the surveillance of AMR during treatment. Studies propose that the combination of DSA testing with other biomarkers such as dd-cfDNA, GEP as well as imaging and clinical assessment may allow for effective rejection surveillance and may in future allow more targeted use of EMBx (62).

Consequently, DSAs have also been investigated as potential markers for CAV risk stratification and surveillance. Baudry and colleagues (63) monitored DSAs at monthly intervals from one-month post-transplant through to twelve months and demonstrated that *de novo* DSAs with complement-binding activity (C3d positive) were associated with a significantly higher risk of severe CAV and mortality up to five years later, with complement-fixing ability appearing to be a key factor in clinical outcome. A further study identified a subgroup of HTx patients that may benefit from the identification of DSA or C4d deposition in the graft endothelium. Patients with C4d positivity had an increased risk of developing CAV compared with those without C4d deposition (*P* = 0.03), and DSA positivity was associated with a shorter mean time to CAV development than a DSA-negative result. CAV severity was similarly affected by DSA status, with 75% of DSA-positive patients who developed CAV progressing to severe CAV, compared with 50% of DSA-negative patients (64). Taken together, current evidence suggests that DSA positivity alone is neither sufficient nor specific for the diagnosis of AMR or CAV; however, the presence of DSA and complement split products identifies a higher-risk subgroup warranting closer surveillance. Incorporating DSA testing alongside complement-binding assays and other biomarkers such as dd-cfDNA may improve risk stratification and guide the timing and intensity of clinical intervention in HTx recipients.

#### 2.2.2 Donor-derived cell-free DNA (dd-cfDNA)

dd-cfDNA refers to fragments of the donor's DNA that are found circulating in the recipient's bloodstream. Levels of dd-cfDNA can always be detected. However, levels rise in proportion to allograft injury as cells undergo apoptosis (programmed cell death), necrosis and release DNA fragments into the plasma (65), or other mechanisms of cell-death resulting in graft injury.

In ACR, dd-cfDNA has received considerable traction as a surveillance biomarker with its high NPV. It has proven to be a sensitive and quantitative biomarker, particularly for low-risk patients with ACR. Agbor-Enoh et al. (66) identified a sensitivity and specificity of above 80% and a NPV of 99.2%. This led to the hypothesis that using dd-cfDNA levels for ACR surveillance could lead to the reduction of 80% of EMBx.

Likewise, Khush, Patel et al. (10), highlighted that dd-cfDNA was able to differentiate between no rejection and ACR grade ≥2R (*P* = 0.004) and a NPV of 97%. Similar elevations in dd-cfDNA had been observed in the setting of AMR. A three times increase in patients with pAMR2 to patients with pAMR0 biopsies and a NPV of 98.6% was also reported. Kim et al. (65) reported significantly elevated levels of dd-cfDNA levels relative to no rejection across all across AMR grades, pAMR1 (H+) (histology features with negative immunostaining) pAMR1 (I+) (positive immunostaining with unremarkable histology) as well as pAMR2 (both positive) all resulting in *p* < 0.001 (Table 2A).

**Table 2A:** Summary of key studies evaluating donor-derived cell free DNA (dd-cfDNA) for rejection and CAV surveillance after heart transplant.

Study	Rejection Subtype	Quantification of % dd-cfDNA	Sample time-point	Population	Key finding
Agbor-Enoh et al. (66)	ACR/AMR	Shotgun sequencing (Threshold of ≥0.25% dd-cfDNA used)	Serial samples over median 17.7 months follow-up (∼8 EMBx/patient); primary performance analysis restricted to samples/EMBx ≥28 days post-tx.	Multicenter (5 sites). 165 HTx patients 44% Black patients, 49% White, 4% Asian and 3% Other.	∼81% of EMBx potentially avoidable. Sensitivity & specificity >80%; NPV 99.2%;
Khush et al. (10)	ACR/AMR	Targeted next-generation sequencing assay (Threshold of 0.2% dd-cfDNA used)	Serial samples from 55 days to >5 years post-tx (81% of samples were drawn in year 1, 13% in year 2).	D-OAR patients, 740 HTx patients across 26 US centers (demographics shown reflect the reference/no-rejection subgroup, *n* = 676): 70% White, 17% Black, 7% Hispanic, 3% Asian, 3% Other. Separate high-AMR-risk sub study: 33 patients (110 samples), sampled from 14 days post-tx.	Overall (0.2% threshold): 44% sensitivity, 80% specificity, NPV 97.1%. High-AMR-risk cohort: dd-cfDNA ∼3-fold higher in AMR vs. non-AMR (0.50% vs. 0.16%, *P* = 0.004); 88% sensitivity/62% specificity.
P.J. Kim et al. (67)	AMR	Targeted amplification NGS assay Continuous %dd-cfDNA used; thresholds of 0.20% and 0.50% evaluated	Prospective study. Samples collected 2017–2022, up to 5 years post-tx. dd-cfDNA paired with EMBx if drawn same day or up to 14 days prior; primary performance analysis restricted to samples/EMBx ≥55 days post-tx (*n* = 8,851 paired samples).	59 centers (67 enrolling; 59 with fully verified data). 2,240 HTx patients. 65.8% White, 21.6% Black, 12.6% Other/unknown.	dd-cfDNA rises significantly with AMR and its severity (non-AMR 0.06% vs. AMR 0.57%, *p* < 0.001; pAMR2/3 median 2.39%), with the 0.50% threshold giving strong specificity (92.8%, LR + 7.29)
Böhmer et al., (27)	ACR/AMR	rd-cfDNA and DF measured separately via ddPCR. Absolute dd-cfDNA calculated (Absolute dd-cfDNA levels = rd-cfDNA x DF)	Samples collected over a 4-year enrolment period. 1,007 paired samples taken concurrently with EMBx during 1st year post-tx. Additional sample taken 3 days post initiation of treatment after AR episode. First 14 days post-tx excluded from AR analysis.	3 sites. 94 HTx patients. 24 paediatric and 70 adults.	Absolute dd-cfDNA also showed significantly better discrimination of symptomatic rejection than DF (AUC = 0.87 vs. 0.75, *p* = 0.02)
Kim et al. (68)	ACR/AMR	SNP-based multiplexed PCR-NGS assay. Two metrics derived: dd-cfDNA and DQS. Thresholds dd-cfDNA >0.26% or DQS >18 cp/mL (either exceeded = positive)	Cross-sectional study. 808 Samples collected concurrently with EMBx. Samples taken prior to 28 days post-tx excluded.	Two centers. 187 HTx patients, 49.2% White, 21.9% Hispanic, 12.8% African American, 10.2% Asian, 5.9% Other.	DQS with dd-cfDNA% (2TA) has better detects AR over dd-cfDNA% alone
Holzhauser et al. (69)	CAV	Targeted amplification NGS assay. ≥0.12% dd-cfDNA threshold.	Cross-sectional study. Single time point sampling, inflammatory and angiogenesis markers taken concurrently. DSA results taken from medical records of sample from same day.	Single center. 65 patients screened; 58 HTx patients analyzed (7 excluded due to unknown CAV status) ≥2 years post-tx	Higher dd-cfDNA level was associated with CAV (*P* = 0.047)
Alharethi et al. (70)	CAV	Targeted amplification NGS assay. No threshold derived.	Mixed retrospective/prospective study.: retrospective biobank samples (INSPIRE registry). prospective enrolment of patients, samples collected at enrolment and after 6 months.	Two center study. 69 HTx patients, 101 plasma samples. Population 81% White.	No association with CAV presence/severity (*P* = 0.25, *P* = 0.52, respectively)
Jiménez-Blanco Bravo et al. (55)	CAV	SNP-NGS assay. Threshold 0.7% dd-cfDNA.	Prospective study. Sample taken concurrently with coronary angiogram in patients >1 year post-tx (median follow-up 10.9 years).	One center. 94 HTx patients. Exclusion of <18 and >80. Exclusion of pt with history of ACR or AMR prior 6 months before sample.	dd-cfDNA showed no significant difference across CAV severity (*P* = 0.0535) or presence (*P* = 0.059)

The different populations of these three studies should be noted (Table 2A), Agbor-Enoh et al. (66), report a higher proportion of Black patients within their cohort compared to other studies. Black patients' dd-cfDNA levels are reported to be higher peri-transplant, and Black patients also show increased rates of rejection (71, 72). The variation in population could have influenced the derived thresholds. Therefore, the outcomes of the results when applied to other ethnicities could vary. Kim et al. (65) note there may have been a recruitment bias, with a higher number of patients with previous episodes of AMR being included. While Khus, Patel, et al. (10) note that their population may have been more clinically stable than other cohorts. With these population differences in mind, PPV and NPV values will differ due to variation of AR prevalence in these differing populations.

Böhmer et al.'s (27) results also showed a significant increase in dd-cfDNA levels compared to controls in both ACR and AMR cases. This study differs from those discussed above in that they quantified the absolute amount of dd-cfDNA using droplet digital PCR, rather than expressing it as fraction of total cfDNA (donor fraction, DF). The results showed improved diagnostic accuracy using absolute dd-cfDNA levels compared to DF alone (AUC 0.87 vs. 0.75) in asymptomatic vs. symptomatic rejection. This allowed for more accurate assessment of dd-cfDNA, as the recipient's own cfDNA levels can vary due to several factors including infection, exercise, recipients' age and sex. These can diminish or augment the DF regardless of injury to the graft (26). This method highlights the true donor DNA signal, one that is not altered by changes in total circulating cfDNA. Kim et al. (68) took a similar approach but instead combined dd-cfDNA fraction and absolute quantity (via a donor-quantity score) into a two-threshold algorithm and compared the results against dd-cfDNA% alone. This combined approach showed better diagnostic accuracy, though it did not reach statistical significance (AUC 0.881 vs. 0.865, *P* = 0.81). However, absolute dd-cfDNA quantification also associates significant limitations due to the lack of standardized thresholds and assay platforms, and the absolute copy-number values vary depending on the quantification method (ddPCR vs. SNP-based NGS) and pre-analytical variables (plasma processing and extraction) (26).

The ISHLT has highlighted the potential role of dd-cfDNA in the context of a pandemic, where home testing could reduce patient exposure to infection due to less need for in-person hospital visits (7). Despite this, EMBx still remains the diagnostic gold standard for AR. dd-cfDNA is already routinely used in some transplant centers, where it can identify patients at minimal risk of rejection and support a shift towards a biomarker-guided biopsy approach. This mitigates the risk of EMBx and reduces procedural burden, patient anxiety (H. Kim et al., (29), and healthcare resource use associated with surveillance biopsies. In the future, this approach may allow for EMBx to be reserved for patients at higher clinical risk.

The potential utility of dd-cfDNA in CAV is now also being explored. Holzhauser et al. (69) examined the relationship between dd-cfDNA and CAV, identifying that patients with elevated dd-cfDNA levels (≥0.12%) had a significantly higher prevalence of angiographically confirmed CAV compared with those with lower levels of dd-cfDNA (63% vs. 35%, *p* = 0.047). Although these findings suggest that dd-cfDNA may reflect ongoing endothelial and vascular injury associated with CAV, several limitations mitigate these conclusions.

Alharethi et al. (70) used the same commercial assay as Holzhauser et al. (69), to evaluate 69 adult HTx recipients. In contrast, no correlation was found between dd-cfDNA concentration and the presence or severity of CAV (*P* = 0.52 and *P* = 0.25, respectively). The authors suggested this finding may reflect the characteristics of the study cohort, as most patients in the study were asymptomatic and had relatively preserved graft function, which may have limited the ability to detect an association between dd-cfDNA and advanced disease.

Jiménez-Blanco Bravo et al. (55) evaluated 94 HTx recipients and found no significant difference in dd-cfDNA levels across CAV severity, nor between patients with stable vs. progressive disease (*P* = 0.059). As well as an AUC-ROC of 0.38, below the level expected by chance (15). This suggests that dd-cfDNA is unreliable for CAV in this population.

The three studies looking at dd-cfDNA in CAV do not agree, with only Holzhauser et al. (69), reporting a significant association. The studies also share the same limitations. Each only took one or two measurements, at a single time point. Therefore, none of them can show how dd-cfDNA changes as CAV starts, progresses, or regresses. All three had small sample sizes, which weakens the results further. None measured absolute dd-cfDNA, only fractional levels, which can be skewed by a patient's own cfDNA regardless of graft injury. Even the positive result from Holzhauser et al. should be read with caution given these limits. Right now, we cannot say for sure whether dd-cfDNA is linked to CAV or not.

The studies differ in methodology as well. Holzhauser et al. (69) used a fixed threshold (≥0.12%) taken from AR studies, not one built for CAV. Jiménez-Blanco Bravo et al. (55) also used a different assay to the other two. These differences in threshold, assay, and design, plus the small and varied patient groups, make it hard to compare the studies directly. This may explain why the results conflict. For now, no firm conclusion can be drawn on dd-cfDNA's use in CAV. Larger, prospective studies using the same assay and threshold are needed before its role is clear.

Overall, dd-cfDNA gives specific, real-time information on graft injury. Its potential use in ACR and AMR is mentioned in the ISHLT guidelines (7). Its role in CAV, though, is still unclear, with conflicting results across the three studies available. It also cannot tell us the cause of the injury. Levels rise in ACR, AMR, and CAV alike. This limits its use as a standalone biomarker.

#### 2.2.3 Gene expression profiling (GEP)

A gene assay has been developed following identification of genes associated with immune activation in relation to ACR pathophysiology in HTx recipients. This assay was derived from the analysis of 4,917 peripheral blood mononuclear cell (PBMC) and EMBx paired samples, from which eleven genes were identified and a further nine genes were included to normalize gene expression levels and ensure sample quality. The eleven genes encode proteins with varied functions, including T-lymphocyte activation, migration, and function, along with steroid responsiveness and hematopoiesis. Dependent on expression levels of the identified genes, the assay generates a numerical score ranging from 1 to 40, with a higher score reflecting a higher probability of rejection (11).

In the multicenter CARGO II study, GEP demonstrated a high NPV of 98.1% or 100% dependent on the threshold score used. This indicates a high diagnostic value in ruling out low-risk patients, potentially providing a reduction in surveillance biopsies (22). To date, no peripheral blood GEP test has been validated for AMR, however studies evaluating histological samples and biopsies via RNA extraction and microarray assays identified AMR specific molecular signatures such as endothelial activation, Natural Killer cells and interferon-γ (73). However, this still requires EMBx and therefore does not qualify as a non-invasive biomarker.

There is only one study to date examining GEP in the context of CAV; Yamani et al. (74) found that patients with CAV demonstrated significantly higher scores compared with those without CAV (*p* = 0.0002). However, no analysis was carried out comparing scores across CAV severity (CAV_0_-CAV_3_).

Between the CAV and ACR study, which used the same assays, it is important to note that the GEP assay has only been validated for ACR; no CAV specific genes set have been validated yet. This may be difficult as the pathophysiology of CAV is still unknown and for this reason may be difficult to derive specific genes. Furthermore, the ACR threshold was derived from large multicenter cohorts and the CAV findings from a small single center cohort (Table 2B). This dissimilarity on scale further limits the extent in which the CAV derived threshold can be considered directly comparable or as robust as the established ACR threshold.

**Table 2B:** Summary of key studies evaluating gene expression profiling (GEP) for rejection and CAV surveillance after heart transplant.

Study	Rejection subtype	Genes Identified	Sample time point	Population	Key findings
Crespo-Leiro et al. (11)	ACR	11 genes identified for rejection. 9 genes identified for control and normalization. ≥34 score cutoff identified	Samples collected prior to EMBx. 1,579 samples concurrent with EMBx and GEP scores.	17 centers 499 HTx patients. 89% White, 3% African American, 2% Asian, 2% Hispanic, 4% Other Patients required to be ≥55 days post-Tx for inclusion.	High false-positive rate (89.8%) NPV 98.1%–100% (threshold dependent)
Loupy et al. (73)	AMR	Six separate biological gene sets. NK cell genes (*n* = 11), DSAST genes (*n* = 69), GRIT/interferon-γ response genes (*n* = 114), macrophage transcripts (*n* = 160), AMR gene set (*n* = 97), and heart parenchymal transcripts (*n* = 2,434). Multi-panel molecular signature used to derive six separate gene-set scores	Case-control design: 55 AMR patients (71 biopsies) matched with 55 non-AMR controls (71 biopsies; 42 normal, 29 with ACR GEP patterns identified in AMR + ve biopsies.	Multicenter (4 sites). 617 HTx patients. No race/ethnicity reported. Case-control design: 55 AMR patients (71 biopsies) matched with 55 non-AMR controls (71 biopsies; 42 normal, 29 with ACR	Tissue-based transcripts could precisely distinguish AMR from non-AMR cases across 4 gene transcript sets (*P* < 0.0001), and results were reproducible and validated in an independent external cohort (Edmonton, Canada; gene set enrichment *P* = 0.01).
Yamani et al. (74)	CAV	No CAV specific genes identified. Genes used were specific to rejection and control genes. ≥30 score cutoff identified	Single time point sample. Blood sample taken same day as EMBx. Score derived from blood sample.	Single center. 69 HTx patients. 20 patients with CAV.	GEP score higher in CAV vs. controls (*P* = 0.0002) Limited sample size, no analysis across CAV severity

#### 2.2.4 MicroRNAs (miRNAs)

miRNAs are small, non-coding RNA molecules that play a key regulatory role in post-transcriptional gene expression. Studies have demonstrated their utility as a rule-out tool with high NPV in patients with ACR and AMR (12).

Elevated levels of miR-142-3p and miR-101-3p have shown to have high discriminatory potential between patients with ACR and no rejection within the first 3–6 months of transplant (75). miRNAs have also been shown to be differently expressed in ACR and AMR, offering potential differentiation between both forms of rejection via a non-invasive test. The GRafT researchers identified 12 different types of miRNAs associated with ACR and 17 associated with AMR. A clinical score ranging from 0 to 100 was developed to aid in the grading of rejection. At the threshold of ≥65, the ACR score showed 86% sensitivity, 78% specificity and an NPV of 98%, for AMR the score threshold showed 82% sensitivity, specificity of 84% and NPV of 97% (12).

Their relevance to CAV has emerged from the hypothesis that certain miRNAs are involved in inflammatory and vascular remodeling pathways, central features of CAV pathophysiology, and may serve as biomarkers of vascular injury. Studies have demonstrated associations between specific miRNAs and CAV. Neumann et al. (76), performed miRNA profiling in patients with confirmed high-grade CAV and identified that plasma levels of both miR-628-5p and miR-155 were significantly elevated compared with controls (*p* = 0.001 and *p* = 0.028, respectively). A threshold value for miR-628-5p above 1.336 predicted CAV with 72% sensitivity and 83% specificity, representing a potential non-invasive miRNA biomarker for CAV.

More recently, González-Torrent et al. (13) carried out a two-phase single-center study examining 70 samples to identify and validate a miRNA signature for CAV. Nine miRNAs were found to be significantly associated with CAV, from which a three-miRNA signature (miR-223-3p, miR-23a-3p, miR-23b-3p) was identified. When combined with donor age, this signature demonstrated high discriminatory diagnostic potential for CAV vs. non-CAV, with a negative predictive value of 97%, sensitivity of 87%, and specificity of 81%. Granting these findings are promising the absence of standardized miRNA panels or assay platforms, together with small sample sizes and single-center designs to limit the power of these studies. Multicenter validation is required before miRNAs can be utilized in routine clinical investigations. Nevertheless, miRNAs represent an emerging and potentially discriminatory biomarker between rejection subtypes.

#### 2.2.5 Molecular microscope diagnostic system (MMDx-Heart)

Although the Molecular Microscope Diagnostic System (MMDx-Heart) is a tissue-based diagnostic requiring an invasive EMBx, its inclusion in this review is warranted because it has emerged as a molecular reference standard against which some non-invasive biomarkers are increasingly calibrated and validated.

MMDx applies microarray-based transcriptomic profiling to endomyocardial tissue obtained from an invasive EMBx, using machine learning algorithms to identify different patterns of rejection (77). MMDx considers rejection and injury as continuous gradients of intensity rather than binary categories. Across cohorts, MMDx consistently reclassifies a substantial minority of biopsies, most often in the direction of increased antibody-mediated rejection (AMR) detection.

In a real-world series of 228 paired MMDx and EMBx specimens, agreement with histology was moderate (84%, *k* = 0.48) but systematically discordant, with MMDx calling rejection in 32 of 37 discordant cases (78). In a prospective single-center study of 351 biopsies, MMDx identified AMR twice as often as histology (14.8% vs. 6.0%; OR 3.26, 95% CI 1.65–6.45), including in DSA-negative biopsies (8.4% vs. 1.7%) (79). MMDx also integrates coherently with blood-based biomarkers showing an agreement with dd-cfDNA for any rejection of approximately 76% (80).

MMDx is mostly framed as a tool providing additional information to histology, and its principal value is to resolve ambiguous or discordant scenarios. Several caveats are associated with its technique, as there is no independent gold standard, so the higher rejection detection rate cannot be equated with improved specificity (79). Furthermore, the assay requires a dedicated fresh biopsy fragment in stabilizing solutions and centralized microarray processing, limiting availability, and turnaround. Alternative targeted platforms applicable to routine formalin-fixed paraffin-embedded tissue have shown ROC-AUCs of roughly 0.81–0.85 for AMR and acute cellular rejection and may broaden access to intra-graft molecular diagnostics (81).

## 3 Emerging biomarkers and future directions

Torque Teno Virus (TTV) is a small, single-strain DNA virus with near-universal prevalence (>90%) with no known pathogenicity (82). Its replication is primary controlled by T-cell mediated immunity, and its plasma viral load reflects the host´s functional immune status. The TTV viral load can help guide immunosuppression tapering and through more appropriate dosing reduce unnecessary malignancy and infection-related morbidity and mortality while also improving patient quality of life (83).

In recent years, there has been a significant development in “omics” technology. These advanced scientific tools enable the analysis of biological molecules on a massive scale. The use of genomics, transcriptomics, proteomics and metabolomics can be combined in a multi-omics approach that will offer a comprehensive perspective in the detection of the different immunological pathways involved in CAV detection, as well as ACR and AMR. High-throughput multi-omics and multi-biomarker panels might be useful to identify profiles affecting micro and macrovascular disease, having a role in the pathophysiological process of CAV.

The use of endothelial injury markers like circulating apoptotic endothelial cells and apoptotic endothelial microparticles might reflect the endothelial damage in patients with CAV capturing early stages not detected by conventional biomarkers (84). Furthermore, lipid markers like lipoprotein A, and vascular markers like ET-1 and VEGF, might be associated with CAV in small rates (85).

The field is moving toward multi-biomarker and multi-omics panels integrated with advanced analytics rather than single-biomarker approaches. However, little information is available at the moment integrating multiple biomarkers in CAV.

Critical unmet needs include prospective validation of candidate biomarkers in large multicenter cohorts, standardization of assay platforms, determination of optimal sampling intervals, and integration of circulatory biomarkers with non-invasive imaging to created comprehensive surveillance frameworks that could reduce reliance on invasive angiography in these patients.

Ideally, we would be able to identify a single blood-based panel of tests which could prospectively identify onset of each of these conditions at an early timepoint and before significant damage to the graft has occurred. Critically, we require information to identify the relevant pathological processes at play enabling early and targeted clinical intervention. Furthermore, such biomarkers would ideally enable personalized dosing for immunosuppression, providing true personalized care for HTx recipients.

Future research priorities should include outcome-based evidence. dd-cfDNA and GEP have been adopted largely based on diagnostic accuracy, and randomized trials are still required to show that biomarker-based surveillance produces graft survival and mortality at least equal to biopsy, and that treatment decisions guided by these tests improve outcomes. Additionally, the tests must cover what is currently missing. They do not reliably separate cellular from AMR and perform poorly for CAV and subclinical injury; refinements such as cell-free DNA fragment analysis, rejection-specific microRNA panels, and EVs are promising but need validation in large, independent cohorts. AI-based risk prediction models might be able to offer better results by combining multiple biomarkers.

## 4 Clinical implementation

Non-invasive biomarker surveillance involves a paradigm shift from “biopsy-first” to “biomarker-guided biopsy”. A decision about when to start testing, how to sequence assays, and what triggers confirmatory biopsy needs to be considered by the transplant groups considering the current limitations of non-invasive biomarkers.

GEP cannot be used before 55 days post-transplant and requires a lower dose of corticosteroids as they alter the expression of steroid-responsive genes; dd-cfDNA reaches a stable baseline at approximately 14 days, and some centers begin testing at 3–4 weeks, with programs using the combined GEP and dd-cfDNA tests generally after 55 days (69). Many centers pair the first non-invasive test with a same-day surveillance EMBx for internal calibration, then taper biopsies according to individual and program-level rejection risk, clinician comfort, and testing resources. The most experienced programs transition acceptable-risk recipients fully to GEP/dd-cfDNA as early as 8 weeks, reserving biopsies for abnormal non-invasive biomarker results, symptoms, or imaging evidence of graft dysfunction.

Implementation is not yet standardized, and this is the principal barrier to guideline-level endorsement. Protocols vary significantly by center in biopsy frequency, thresholds applied, and the handling of discordant non-invasive biomarker results. The currently defined thresholds offer limitations depending on age and body size, and underlying conditions such as infection, blood transfusions, pregnancy or other transplanted organs are also a limitation (67). No randomized clinical trial has yet compared or validated an EMBx based on a biomarker-based pathway. The SHORE registry, including approximately 2,300 recipients monitored with GEP/dd-cfDNA from within 3 months of transplant vs. historical controls, is the largest source of forthcoming implementation relevant outcome data (86). Further prospective trials are needed to generate evidence to standardize the use of non-invasive biomarkers and be fully endorsed by the guidelines.

## 5 Limitations

This review has several methodological limitations inherent to its narrative design. The literature search was not systematic, no protocol was registered, and no formal risk-of-bias or quality appraisal of included studies was performed. Selection and citation bias therefore cannot be excluded, and studies reporting positive or novel biomarker associations are likely over-represented relative to negative or confirmatory work. No quantitative synthesis or meta-analysis or diagnostic accuracy was attempted, so the sensitivities, specificities, and predictive values quoted throughout are reported as published rather than pooled and are not directly comparable across studies that used different assay platforms, rejection definitions, and reference standards. Reported predictive values are additionally dependent on the prevalence of rejection in the source cohort, which is generally low in contemporary surveillance populations and inflates negative predictive value. Finally, this is a rapidly moving field with several registries and interventional trials in progress, and some conclusions drawn here will require revision as those data mature.

Important limitations also apply to the evidence base itself. Most candidate biomarkers beyond dd-cfDNA and GEP have been evaluated in single-center, retrospective, or modestly sized cohorts, often with case-control designs that overestimate discriminative performance, and with heterogenous or absent cardiac-specific thresholds.

## 6 Conclusion

This review summarizes the current available evidence in the field of non-invasive biomarkers in HTx recipients on ACR, AMR, and CAV. The use of DSA is an established, guideline-endorsed way to investigate patients suspected clinically or by EMBx to suffer from AMR, and it should be used for regular monitoring in all patients after HTx. BNP/NT-proBNP and troponin are graft-specific biomarkers, and their use in HTx surveillance is only recommended to identify patients for further evaluation. GEP and dd-cfDNA have the best evidence and practically defined thresholds, and their use can best be described as “rule-out” for rejection with a high negative predictive value in a selected population. Combining dd-cfDNA and GEP seems to offer additional surveillance accuracy.

Key advances in the field have been made in the use of non-invasive biomarkers for ACR and AMR. However, the current research and available data for CAV is very limited, and further research focusing on the early identification and immunological mechanisms of this complication is needed.
